# Decreased pre-surgical CD34^+^/CD144^+ ^cell number in patients undergoing coronary artery bypass grafting compared to coronary artery disease-free valvular patients

**DOI:** 10.1186/1749-8090-7-2

**Published:** 2012-01-03

**Authors:** Santiago Redondo, Álvaro González-Rocafort, Jorge Navarro-Dorado, Marta Ramajo, Mihail Hristov, Antonio Gordillo-Moscoso, Fernando Reguillo, Manuel Carnero, Jose Martinez-Gonzalez, Enrique Rodríguez, Christian Weber, Teresa Tejerina

**Affiliations:** 1 Department of Pharmacology, School of Medicine, Universidad Complutense, Madrid, Spain; 2 Service of Hematology, Hospital Nuestra Señora de Sonsoles, Ávila, Spain; 3 Service of Cardiac Surgery, Hospital Clinico San Carlos, Madrid, Spain; 4 Institut für Prophylaxe und Epidemiologie der Kreislaufkrankheiten, Klinikum der LMU, Munich, Germany; 5 Department of Clinical Research, Universidad Autónoma de San Luis Potosí, San Luis Potosí, Mexico; 6 Cardiovascular Research Center (CSIC-ICCC), Hospital de la Santa Creu i Sant Pau, Barcelona, Spain

**Keywords:** cardiac surgery, endothelial progenitor cells, coronary artery disease

## Abstract

**Background:**

Cardiovascular disease has been linked to endothelial progenitor cell (EPC) depletion and functional impairment in atherosclerosis and aortic stenosis. EPCs may play a pivotal role in vascular grafting. However, the EPC depletion in coronary artery bypass grafting (CABG) patients has not been compared to coronary artery disease-free valvular replacement patients with aortic stenosis.

**Methods:**

We aimed to assess the basal number of CD34^+^/KDR^+ ^and CD34^+^/CD144^+ ^cells in CABG patients, compared to aortic stenosis valvular replacement patients. 100 patients (51 CABG and 49 valvular surgery ones) were included in the present study. All CABG or valvular patients had angiographic demonstration of the presence or the absence of coronary artery disease, respectively. Numbers of CD34^+^/KDR^+ ^and CD34^+^/CD144^+ ^were assessed by flow cytometry of pre-surgical blood samples.

**Results:**

We found a lower number of CD34^+^/CD144^+ ^cells in CABG patients compared to valvular patients (0.21 ± 0.03% vs. 0.47 ± 0.08%), and this difference remained statistically significant after the *P *was adjusted for multiple comparisons (*P *= 0.01428). Both groups had more EPCs than healthy controls.

**Conclusions:**

Pre-surgical CD34^+^/CD144^+ ^numbers are decreased in CABG patients, compared to valvular patients with absence of coronary disease.

## 1. Background

Atherothrombosis is the leading global cause of morbimortality [1]. A growing burden of experimental and clinical evidence highlights the importance of endothelial progenitor cells (EPCs) in the pathogenesis of this disease [2,3]. EPCs originate in the bone marrow and migrate in response to ischemic stimuli, especially stromal-derived factor (SDF-1) [4]; they differentiate towards the endothelial lineage or support local angiogenesis by resident endothelial cells. In their differentiation process, EPCs possess the early markers CD34 and KDR and acquire further markers of endothelial commitment (such as CD144, also known as VE-cadherin) [2].

Several studies describe a lower number of CD34^+^/KDR^+ ^cells in patients with coronary artery disease (CAD); these low numbers seem to be inversely correlated with cardiovascular risk factors [4]; moreover, this EPC decrease has been directly linked to CAD [5] and to precede future cardiovascular events [6]. Lower CD34^+^/CD144^+ ^numbers have been described in atherosclerotic patients, as well as the role of statins to differentiate CD34^+^/KDR^+ ^to CD34^+^/CD144^+ ^[7].

Other reports, however, suggest a higher number of EPCs in atherosclerosis [8]. A population-based study shows a direct correlation between CD34^+^/KDR^+ ^and Framingham risk factors [9]. In addition, long-term statin treatment has been related to a reduced number of CD34^+^/KDR^+ ^in patients with CAD [10]. This discrepancy may be explained by the possible oscillation of cell number depending on the vascular disease state, which depends on the intensity of the ischemia [11]. Higher apoptosis and lower viability have been observed in cultured EPCs from atherosclerotic patients, especially diabetics [12,13]. High glucose concentrations impair adhesive properties of EPCs under flow conditions [14]. The recent clinical evidence suggests a role of bone marrow-origin EPC exhaustion in advanced atheroscelrotic cardiovascular disease [15]. However, EPC exhaustion has equally been described in degenerative aortic stenosis and it may mediate endothelial damage in this disorder [16]. Both CABG and valvular replacement surgery dramatically increase the number of EPCs, however these EPCs seem to be functionally impaired after both interventions [17]. The question arises whether CABG with advanced atherosclerotic disease and valvular replacement patients with aortic stenosis and angiographic abscence of coronary lessions possess different EPC levels. Thus, we aimed to assess the number of CD34^+^/CD144^+ ^and CD34^+^/KDR^+ ^cells in the peripheral blood of patients undergoing coronary artery bypass grafting (CABG) with angiographic demonstration of CAD, compared to valvular surgery patients with angiographic demonstration of absence of CAD. The ultimate goal was to elucidate which vascular disorder involves a deeper alteration of EPC numbers.

## 2. Methods

### 2.1. Clinical research design

Patients were recruited from the Service of Cardiac Surgery (Hospital Clinico San Carlos, Madrid, Spain). Coronary angiography was performed in all the patients before inclusion in each group. Included valvular patients had aortic stenosis and abscence of significant coronary stenosis. Patients who suffered CAD and aortic stenotic valvular disease and were operated for both diseases at the same time were equally *a priori *excluded, since we aimed to compare ischemic patients with the angiographically proven presence or absence of coronary artery disease. The study was designed for pre-surgical blood samples. Thus, for logistic disposability of the flow cytometer, consecutive CABG or valvular patients were included once a week, given that the cytometer was reserved every Monday. The n (minimum of 45 per group) was calculated based on the variability of EPC number in a cohort of healthy subjects where this parameter was assessed by using the same laboratory protocol and flow cytometer [18]. These numbers of patients were recruited from May 2007 to May 2009. Exclusion criteria were: end-stage renal or liver disease, previous heart surgery, absence of coronariography, cancer and autoimmune disease. The study was approved by the local Ethical Committee and was conducted according to the declaration of Helsinki. All patients gave written informed consent. As a comparative healthy control group, we included 27 healthy volunteers from the Bosch Company (Madrid, Spain). For this healthy cohort, all cardiovascular risk factors (smoking, obesity, hypertension, hypercholesterolemia, diabetes and physical inactivity) were considered as exclusion criteria.

### 2.2. Basal characteristics of the study groups

The primary objective of the study was to compare the number of CD34^+^/KDR^+ ^and CD34^+^/CD144^+ ^cells in the pre-surgical blood of CABG and aortic stenosis valvular patients, respectively. However, in addition to coronary obstruction [5], other parameters have been shown to affect EPC number: age, sex, body mass index, smoking, alcohol, dyslipidemia, diabetes, antiplatelet drugs, antihypertensive, antidiabetic, hypolipidemic drugs [10] and euroSCORE cardiac surgery risk stratification [19]. Therefore, they were also compared between both groups and taken into account for the statistical analysisis. The euroSCORE was calculated as a logistic index.

### 2.3. Definitions

Diabetes mellitus was defined according to the criteria of the American Diabetes Association as fastin serum glucose above 126 mg/dl, or the use of antidiabetic drugs. Dyslipidemia was defined as fasting total serum cholesterol > 200 mg/dl, LDL > 100 mg/dl, triglycerides > 180 mg/dl, or the use of lipid-lowering therapy. Myocardial infarction was defined as a transient increase of specific serum markers of myocardial necrosis (CK-MB or troponin T) along with ischemic symptons and/or typical electrocardiographic signs. A coronary vessel was considered as obstructed if luminal obstruction reached 50% (for the main trunk coronary artery) or 75% (for any of the three principal vessels).

### 2.4. Blood collection and flow cytometry

**Figure 1:**
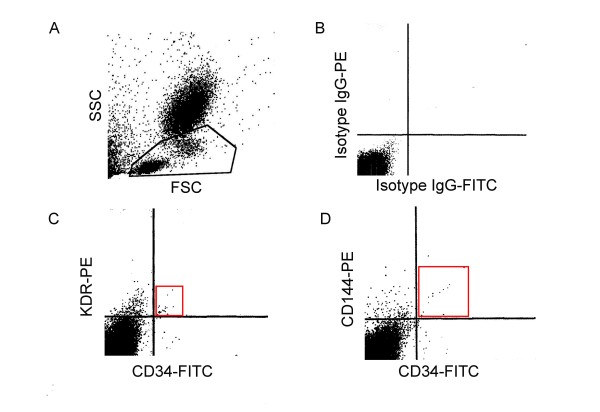
**Percentage of double-positive cells in peripheral blood or cell culture samples**. Panel A: Representative gating strategy. Panel B: Representative cytometry profile for control IgG. Panel C: Representative cytometry profile for CD34^+^/KDR^+^. Panel D: Representative cytometry profile for CD34^+^/CD144^+^.

Percentage of CD34^+^/KDR^+ ^and CD34^+^/CD144^+ ^cells was assessed as described [18]. Blood was taken before the surgical procedure using EDTA-Vacutainer^® ^tubes. Immunostaining was performed immediately. Briefly, 10 μl of respective antibodies were added to each 100 μl of aliquoted blood: mouse IgG_1k _isotype control fluorescein isothiocyanate conjugated (555748, Becton Dickinson, Franklin Lakes, NJ, USA), mouse IgG_1 _isotype control phycoerythrin conjugated (IC002P, R&D Systems, Minneapolis, MN, USA), mouse IgG_2B _isotype control phycoerythrin conjugated (IC0041P, R&D Systems), mouse IgG_1k _anti-CD34 isothiocyanate fluorescein conjugated (555821, Becton Dickinson), mouse IgG_1 _anti-VEGF-R2 (KDR), phycoerythrin conjugated (FAB357P, R&D Systems) and mouse IgG_2B _anti-CD144 (VE-cadherin) phycoerythrin conjugated (FAB9381P, R&D Systems). The blood was incubated for 30 min on ice protected from the light. Red blood cells were lysed by a commercial lysis solution (347691-MSDS-A, Becton Dickinson) for 10 minutes at room temperature. This lysis buffer also acts a smooth fixative. Samples were kept at 4°C protected from the light and cytometric analysis was made within the next 24 hours, which yields a high intra-assay correlation compared to fresh analysis (r^2 ^= 0.8517). This analysis was performed using a FACScalibur flow cytometer (Serial Number E2295, BD, San Jose, CA, USA). Mononuclear cells were selected for the analysis. The events were plotted as linear SSC/FSC and the beams lower left (considered as lymphocytes), and immediately upper right (considered as monocytes) were gated for the analysis, whereas polymorphonuclear cells (with a higher size and complexity) were discarded. A representative gating strategy is shown in Figure 1, panel A. Cytometry technicians were unaware of each patient data group. Analysis was performed using CellQuestPro^®^. Briefly, the quadrants were defined according to the control IgG (the region where positive events were not counted) and they were exported for the analysis of the next two experimental samples (CD34^+^/KDR^+ ^and CD34^+^/CD144^+^). Therefore, the positive CD34^+^/KDR^+ ^and CD34^+^/CD144^+ ^events were noted in a region where no event were detected in the control IgG sample. Representative cytometry profiles are shown in Figure 1, panels B, C and D.

### 2.5. Determination of plasmatic concentration of cytokines

Blood from EDTA tubes was centrifuged at 1500 rpm for 15 minutes. Plasma was extracted and frozen at -40°C. Total levels of TGF-β1 (active plus acid-activatable) were assessed by ELISA (R&D Systems, Minneapolis, MN, USA). This kit proved highly reproducible in a large comparative study of several ELISA kits for TGF-β [20]. Plasma levels of IL-6 and TNF-α were assessed by ELISA (Minneapolis, MN, USA), given that these cytokines have been proven to be deeply altered in cardiac surgery [21]. SDF-1 concentration was measured by ELISA (R&D systems, Minneapolis, MN, USA) from platelet-poor plasma, following the manufacturer's instructions.

### 2.6. Statistics

Data are reported as mean and standard error of the mean (SEM) for continuous variables. Normality was assessed by the Shapiro-Wilk test. Comparison and correlation analyses were performed with Student T-test and Pearson coefficient correlation or the correspondent non-parametric tests. Analysis of variance (ANOVA) was used to test for differences in means across treatment groups, and with significant differences a post hoc HSD-Tukey test was used to compare specific treatment groups. Differences with a *P *value of less than 0.05 were considered statistically significant. Multivariate regression analysis was performed between the primary variable *versus *all clinical variables. The Bonferroni test was used for multiple comparisons. Analyses were conducted with the program R, version 2.0.1 (Institute for Statistics and Mathematics, Wirtschaftsuniversität Wien, Vienna, Austria).

## 3. Results

### 3.1. Clinical and laboratory profile of the patients

**Figure 2:**
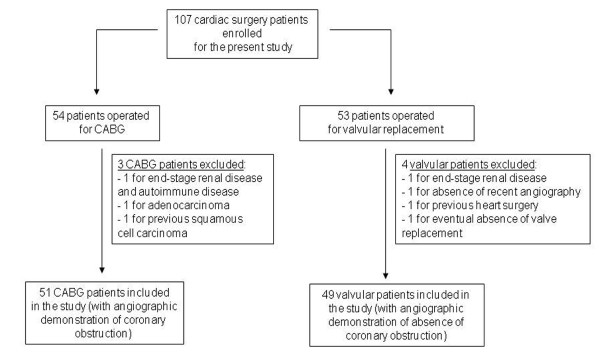
**Flow diagram of the patients**. After all clinical exclusions, 51 patients operated for coronary artery bypass grafting and 49 patients operated for valvular replacement (aortic stenosis) were chosen for the present study.

Following the recruitment protocol stated in Methods, 54 patients operated for CABG and 53 patients operated for aortic stenosis valvular replacement surgery were enrolled for the present study. 3 CABG and 4 valvular patients were excluded, since the second review of their clinical report revealed that they had one or more exclusion criteria after their pre-surgical EPC number was already assessed. This yielded 51 CABG and 49 valvular patients. A flow diagram of the patients is shown in the Figure 2.

As shown in Table 1, CABG patients possessed a significantly higher burden of cardiovascular risk factors (diabetes and dyslipidemia) and medications (antiplatelet drugs, statins, nitrates, oral antidiabetics and insulin). They had lower left ventricular ejection fraction, higher rate of myocardial infarction within 30 days before surgery and a lower logistic euroSCORE risk assessment. This last finding may be explained by the higher mean age and female/male ratio in the valvular group, despite a lower incidence of previous myocardial infarction (Table 1). Given that our hospital is a reference center for cardiac surgery, no data were available for HDL cholesterol and quantification of angina symptoms.

**Table 1:** Clinical and pharmacological characteristics of the patients.

	*CABG*	*Valvular*	*P*
Number	51	49	-
Age	64.65 ± 13.34	72.7 ± 10.4	0.106^¶^
Sex (Women, %)	8 (15.69%)	22 (44.89%)	0.001*
BMI (kg/m^2^)	26.91 ± 0.43	26.51 ± 0.57	0.5726^§^
EF (%)	62.51 ± 1.84	69.2 ± 1.41	0.006^§^
Tobacco	12 (23.53%)	9 (18.37%)	0.698*
Alcohol intake	15 (29.41%)	14 (28.57%)	0.898*
Hypertension	31 (60.78%)	27 (55.10%)	0.486*
Dyslipidemia	31 (60.78%)	16 (32.65%)	0.003*
Diabetes	24 (47.06%)	5 (10.20%)	< 0.001*
ACEI/ARB	25 (49.02%)	19 (38.77%)	0.302*
Beta blockers	16 (31.37%)	9 (18.37%)	0.133*
Calcium blockers	8 (15.69%)	10 (20.41%)	0.539*
Antiplatelet drugs	33 (64.71%)	8 (16.33%)	< 0.001*
Statins	34 (66.67%)	11 (22.45%)	< 0.001*
Nitrates	16 (31.37%)	2 (4.08%)	< 0.001*
Oral antidiabetics	16 (31.37%)	2 (4.08%)	< 0.001*
Insulin	13 (25.49%)	1 (2.04%)	< 0.001*
MI < 30 days	16 (31.37%)	0 (0%)	< 0.001*
EuroSCORE	3.6 ± 0.4194	5.7 ± 0.4	< 0.001*

* = Chi-squared test, ^§ ^= Student's t test, ^¶ ^= U Mann-Whiney-Wilcoxon test

BMI: body mass index; EF: ejection fraction; MI; myocardial infarction

CABG had a significant increase of platelets, monocytes and fibrinogen. Plasmatic levels of TGF-β1, SDF-1, IL-6 and TNF-α remained unchanged, suggesting that none of these plasma markers were specific for angiographic coronary obstruction (Table 2).

**Table 2:** Biochemical and hematological data.

	*CABG*	*Valvular*	*P*
Number	51	49	-
Glucose	108.2 ± 5.12	108.9 ± 6.56	0.4258^¶^
Creatinine	1.2 ± 0.049	1.154 ± 0.045	0.5003^§^
eGFR (ml/min/1.73)	70.24 ± 2.96	65.61 ± 2.96	0.2614
Platelets	240200 ± 94	211100 ± 83	0.0228^§^
Hemoglobin	13.09 ± 0.03	13.76 ± 0.03	0.0762^§^
Leucocytes	8255 ± 298	7525 ± 298	0.0871^§^
Neutrophils	4939 ± 225.6	4534 ± 319.9	0.3008^§^
Monocytes	689.6 ± 36.01	566.1 ± 28.20	0.0085^§^
Lymphocytes	2273 ± 109.8	2122 ± 125.3	0.3667^§^
Total CD34^+^/KDR^+^/μl	8.204 ± 4.01	7.347 ± 2.60	0.0952^¶^
Total CD34^+^/CD144^+^/μl	3.684 ± 0.70	7.694 ± 2.21	0.0217^¶^
Fibrinogen	484.4 ± 20.63	387.7 ± 11.07	< 0.0001^§^
TGF-β1 (ng/ml)	55.73 ± 3.55	52.48 ± 3.46	0.5144^§^
SDF-1 (ng/ml)	3.525 ± 0.11	3.604 ± 0.11	0.6152^§^
IL-6 (pg/ml)	3.152 ± 0.80	2.542 ± 0.29	0.4824^§^
TNF-α (pg/ml)	3.745 ± 0.55	4.099 ± 0.68	0.7216^§^

^§ ^= Student's t test, ^¶ ^= U Mann-Whiney-Wilcoxon test

eFGR: Estimated glomerular filtration rate (ml/min/1.73 m)

In the control cardiovascular risk factor-free group (with absence of angiographic study for ethical reasons), the mean age was 42.78 ± 1.969 (mean ± SEM) and the percentage of women was 35.71%.

### 3.2. CD34^+^/KDR^+ ^and CD34^+^/CD144^+ ^numbers

**Figure 3:**
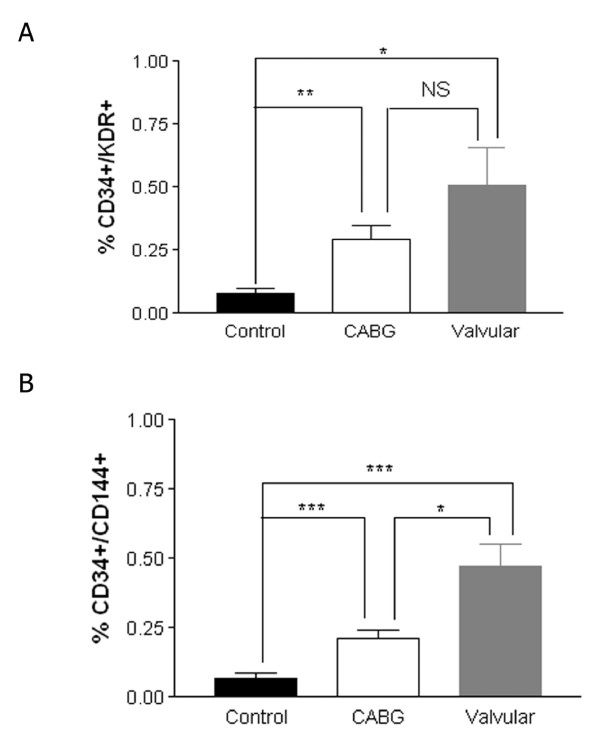
**Abundance of CD34^+^/KDR^+ ^and CD34^+^/CD144^+ ^cells in the pre-surgical peripheral blood of the patients, and healthy controls**. Panel A: abundance of CD34^+^/KDR^+ ^cells. Panel B: abundance of CD34^+^/CD144^+ ^cells.

As shown in Figure 3, panel A, CABG and aortic stenosis valvular patients did not possess a significantly different level of CD34^+^/KDR^+ ^cells, although a decreasing trend was noted for CABG. Figure 3, panel B shows the numbers of CD34^+^/CD144^+ ^cells. Patients ongoing CABG had a lower number of CD34^+^/CD144^+ ^cells compared to valvular surgery patients, in a significant manner. The total numbers of CD34^+^/KDR^+ ^and CD34^+^/CD144^+ ^per μl were equally assessed. As shown in the Table 2, yet again the total number of CD34^+^/CD144^+ ^per μl remained lower in the valvualr group compared to the patients who underwent CABG, whereas CD34^+^/KDR^+ ^remained unchanged. Both groups of patients had higher numbers of CD34^+^/CD144^+ ^cells compared to healthy volunteers without cardiovascular risk factors (Figure 3).

In a subgroup of aortic stenosis valvular patients where echocardiac valvular gradient (mmHg) and valvular area (cm^2^) were available, there was not a significant correlation among these parameters and the abundance of CD34^+^/CD144^+ ^(r^2 ^= 0.021, *P *= 0.41 for mean gradient and r^2 ^= 0.015 and *P *= 0.48 for valve area, respectively).

### 3.3. Effects of clinical and laboratory parameters on CD34^+^/CD144^+ ^numbers

In order to explore whether the different numbers of CD34^+^/CD144^+ ^may be mediated by different clinical and analytical parameters between CABG and aortic stenosis valvular patients, intragroup analysis using these parameters were made. As shown in Table 3, in the CABG group only the presence of dyslipidemia was associated to decreased CD34^+^/CD144^+ ^counts. This suggests that the clinical and laboratory differences between CABG and valvular patients (Tables 1 and 2) are not responsible of the different numbers of CD34^+^/CD144^+ ^cells between these two groups. However, in these non-primary endpoints the *P *values were not adjusted to the number of comparisons and thus should be interpreted in an exploratory manner.

**Table 3:** Intragroup variation of CD34^+^/CD144^+ ^according to different parameters.

	*CABG*	*Valvular*
Sex	Men: 0.2276 ± 0.03 Women: 0.1259 ± 0.05 (P = 0.13)^¶^	Men: 0.4189 ± 0.08 Women: 0.5423 ± 0.15 (P = 0.54)^¶^

EF (%)	< Mean: 0.2492 ± 0.05 > Mean: 0.1722 ± 0.03 (P = 0.21)^¶^	< Mean:0.5375 ± 0.11 > Mean: 0.4419 ± 0.12 (P = 0.46)^¶^

Tobacco	Yes: 0.1583 ± 0.05 No: 0.2300 ± 0.03 (P = 0.35)^¶^	Yes: 0.43 ± 0.12 No: 0.4835 ± 0.09 (P = 0.52)^¶^

Dyslipidemia	Yes: 0.1542 ± 0.03 No: 0.3045 ± 0.05 (P = 0.016)^¶^	Yes: 0.3363 ± 0.08 No: 0.5412 ± 0.11 (P = 0.38)^¶^

Diabetes	Yes: 0.2129 ± 0.05 No: 0.2133 ± 0.04 (P = 0.59)^¶^	Yes: 0.4160 ± 0.29 No: 0.4809 ± 0.08 (P = 0.43)^¶^

Antiplatelet drugs	Yes: 0.2330 ± 0.04 No: 0.1767 ± 0.04 (P = 0.66)^¶^	Yes: 0.1925 ± 0.09 No: 0.5293 ± 0.09 (P = 0.0238)^¶^

Statins	Yes: 0.1947 ± 0.03 No: 0.2500 ± 0.06 (P = 0.42)^¶^	Yes: 0.1925 ± 0.09 No: 0.5293 ± 0.09 (P = 0.02)^¶^

Nitrates	Yes: 0.2856 ± 0.06 No: 0.1800 ± 0.03 (P = 0.21)^¶^	Yes: 0.4250 ± 0.20 No: 0.4767 ± 0.08 (P = 0.90)^¶^

Oral antidiabetics	Yes: 0.1944 ± 0.05 No: 0.2217 ± 0.03 (P = 0.31)^¶^	Yes: 0.8950 ± 0.68 No: 0.4564 ± 0.08 (P = 0.28)^¶^

Insulin	Yes: 0.2954 ± 0.07 No: 0.1850 ± 0.03 (P = 0.19)^¶^	Yes: 0.09 ± 0 (n = 1) No: 0.4823 ± 0.08 (P < 0.0001)^§^

MI < 30 days	Yes: 0.2481 ± 0.06 No: 0.1971 ± 0.03 (P = 0.37)^¶^	Yes: 0.4749 ± 0.08 No: 0 ± 0 (P < 0.0001)^§^

EuroSCORE	Yes: 0.1852 ± 0.03 No: 0.2575 ± 0.06 (P = 0.46)^¶^	Yes: 0.4489 ± 0.16 No: 0.4890 ± 0.09 (P = 0.55)^¶^

Platelets	Yes: 0.2403 ± 0.04 No: 0.1710 ± 0.04 (P = 0.27)^¶^	Yes: 0.3627 ± 0.07 No: 0.6004 ± 0.15 (P = 0.48)^¶^

Monocytes	< Mean: 0.2015 ± 0.03 > Mean: 0.2252 ± 0.05 (P = 0.71)^¶^	< Mean: 0.4668 ± 0.13 > Mean: 0.4821 ± 0.09 (P = 0.48)^¶^

Fibrinogen	< Mean: 0.2411 ± 0.05 > Mean: 0.1645 ± 0.05 (P = 0.09)^¶^	< Mean: 0.3945 ± 0.08 > Mean: 0.4700 ± 0.11 (P = 0.98)^¶^

^§ ^= Student's t test, ^¶ ^= U Mann-Whiney-Wilcoxon test.

### 3.4. Multivariate analysis

We performed a multivariate analysis and the *P *values were adjusted to the number of comparisons. As shown in the Table 4, being CABG or valvular yields a significant association with CD34^+^/CD144^+ ^cell numbers after this analysis was performed (*P *= 0.01428 after Bonferroni adjustment).

**Table 4:** Multivariate analysis (the *P *value was adjusted to the number of comparisons by the Bonferroni test).

	*Estimate*	*SEM*	*P*
CABG/Valvular	-5.5532	2.2252	0.01428
ACEI/ARB	5.6474	2.3756	0.01942
Age	-0.2615	0.0887	0.00402

## 4. Discussion

Our results show a lower basal number of CD34^+^/CD144^+ ^cells in the pre-surgical blood of patients who underwent CABG surgery compared to aortic stenosis valvular replacement patients (Figure 3, panel B). Of note, being CABG or valvular was related to CD34^+^/CD144^+ ^cells even after the *P *value was adjusted to the number of comparisons (Table 4). This suggests that this parameter could be related to angiographic CAD in severe and advanced cardiovascular disease.

The clinical characteristics of our patients merit some comments (Tables 1 and 2); although in these non-primary endpoints the *P *values were not adjusted to the number of comparisons and thus should be interpreted in an exploratory manner. CABG had a larger amount of atherosclerotic risk factors and related medications (Table 1) and higher numbers of monocytes, fibrinogen and platelets (Table 2), which reproduces what was found in other series [22]. This reinforces the broadly accepted idea that atherosclerosis acts as an inflammatory stimulus, a concept supported by the finding of augmented CRP levels in a subgroup of our CABG patients (3.4 ± 1.67 mg/l), In the intragroup analysis we found that only dyslipidemia had a significant effect on CD34^+^/CD144^+ ^numbers in CABG patients (Table 3). Of note, the plasmatic concentration of several cytokines (TGF-β1, SDF-1, IL-6 and TNF-α) in CABG and valvular patients remained unchanged (Table 2). Therefore, these parameters were not specific enough to discriminate between patients with angiographic presence or absence of coronary disease in our study.

The numbers of CD34^+^/CD144^+ ^cells in the peripheral blood of cardiac surgery patients (both CABG and valvular) is higher than reported elsewhere for CD34^+^/KDR^+ ^[5,6], and than the one which was obtained by us in a cohort of healthy volunteers [18]. A population-based study shows that CD34^+^/KDR^+ ^cells and SDF-1 levels are directly associated with Framingham risk factors, whereas this correlation becomes inverse when CD34^+^/KDR^+ ^are compared to vessel injury parameters such as intima-media thickness [9]. Our results show that CD34^+^/KDR^+ ^and CD34^+^/CD144^+ ^from healthy subjects without atherosclerotic risk factors are clearly decreased compared to ischemic non-atherosclerotic patients (Figure 3). Thus, our data support the idea that EPCs are released in response to ischemia and they reach different levels in CABG and valvular patients. These findings agree with the emerging concept that EPCs are released in response to ischemia, being further depleted in advanced cardiovascular disease [9]. This may help to explain why several studies show that these cells are reduced in cardiovascular disease [4-6], whereas they are physiologically mobilized from the bone marrow in response to ischemia [2,3]. The idea of EPC exhaustion in atherosclerosis is supported by recent clinical evidence [15]. However, in the present study this decrease is found in a significant manner only for CD34^+^/CD144^+^, not for CD34^+^/KDR^+ ^(Figure 3). Given that CD34^+^/CD144^+ ^is considered as a more differentiated EPC phenotype [7], it could be postulated that this advanced differentiation may be disrupted in CABG patients due to a longer history of atherosclerotic vascular damage. Lower CD34^+^/KDR^+ ^numbers for atherosclerotic populations described elsewhere [6] were not assessed in patients where CAD was angiographically demonstrated and were candidates for surgical CABG. Our data also show a lower number of CD34^+^/CD144^+ ^cells despite a higher rate of cardiovascular drug consumption (Table 1). In the case of statins, the particular history of CABG patients (long-term, severe and advanced angiographic CAD) may also explain the lack of CD34^+^/KDR^+ ^decrease [10] and subsequent differentiation-related CD34^+^/CD144^+ ^increase [7] which has been related to statins.

In addition to CAD, aortic valve stenosis is associated to endothelial damage and decreased EPC number and function has been described in these patients [16]. A proper EPC function may be essential for the favorable outcome of surgical grafts [23]. Both in CABG and in valve replacement surgery, EPCs are dramatically released in response to surgical stress, although they seem to be functionally impaired [17]. Restoration of a proper EPC function in both surgical conditions may prove a promising therapeutic strategy. Nevertheless, no data were available about the comparative basal levels of EPCs in the pre-surgical blood of CABG and valvular replacement patients. In the present study we show that the number of CD34^+^/CD144^+ ^cells is decreased in the pre-surgical blood of CABG patients, compared to valvular patients with angiographic absence of CAD. Both cardiac surgery patients had a higher abundance of CD34^+^/CD144^+ ^cells compared to healthy controls. This may possess a putative diagnostic and prognostic value.

## Conclusions

This is the first study which demonstrates a relative EPC depletion in the pre-surgical blood of patients undergoing CABG compared to patients undergoing valve replacement, where CAD was angiographically demonstrated or ruled out, respectively. Given that EPCs seem to play an important role in cardiac surgery grafting, this finding may help to improve our understanding for the simulation of this reparatory mechanism. The study of the underlying mechanisms of this EPC depletion in CAD will be the subject of further research efforts.

## Competing interests

The authors declare that they have no competing interests.

## Authors' contributions

SR, AG-R, AG-M, FR, MC, TT designed the study. SR, JN-D, MR, MH carried the laboratory work. SR, AG-R, AG-M, FR, MD collected and analyzed the clinical data. SR, MH, JM-G, ER, CW, TT helped to draft the manuscript. All authors read and approved the final manuscript.
